# *In Utero* Environmental Tobacco Smoke Exposure Alters Gene Expression in Lungs of Adult BALB/c Mice

**DOI:** 10.1289/ehp.10358

**Published:** 2007-09-19

**Authors:** Rodney L. Rouse, Marc J. Boudreaux, Arthur L. Penn

**Affiliations:** Comparative Biomedical Sciences, Louisiana State University School of Veterinary Medicine, Baton Rouge, Louisiana, USA

**Keywords:** allergy, asthma, environmental tobacco smoke (ETS), gene regulation, *in utero*, inflammation, lung, ovalbumin

## Abstract

**Background:**

*In utero* environmental tobacco smoke (ETS) exposure exacerbates initial lung responses of adult mice to ovalbumin (OVA), a common allergen in rodent models of allergic asthma.

**Objective:**

We tested the hypothesis that *in utero* ETS exposure alters expression of genes (including asthma-related and inflammatory genes) in the lungs of adult mice and that this differential expression is reflected in differential respiratory and immune responses to nontobacco allergens.

**Methods:**

Using Affymetrix Mouse Genome 430 2.0 arrays, we examined gene expression changes in lungs of BALB/c mice exposed to ETS *in utero,* OVA, or saline aerosol at weeks 7–8, and OVA sensitization and challenge at weeks 11–15. Data sets were filtered by transcript *p*-value (≤ 0.05), false discovery rate (≤ 0.05), and fold change (≥ 1.5). Differential expression of selected genes was confirmed by polymerase chain reaction (PCR).

**Results:**

Genes differentially expressed as a result of *in utero* ETS exposure are involved in regulation of biological processes (immune response, cell proliferation, apoptosis, cell metabolism) through altered cytoskeleton, adhesion, transcription, and enzyme molecules. A number of genes prominent in lung inflammation were differentially expressed on PCR but did not pass selection criteria for microarray, including arginase (*Arg1*), chitinases (*Chia*, *Chi3l3*, *Chi3l4*), eotaxins (*Ccl11*, *Ccl24*), small proline-rich protein 2a (*Sprr2a*), and cytokines (*Il4*, *Il6*, *Il10*, *Il13*, *Tnfa*) .

**Conclusion:**

The differential lung gene expression reported here is consistent with previously reported functional changes in lungs of mice exposed *in utero* to ETS and as adults to the nontobacco allergen OVA.

The incidence of asthma and allergy has increased dramatically during the past 30 years, primarily in industrialized countries (Eder et al. 2006). Although improved availability and quality of medical care account for some of this increase, the magnitude of the increase surpasses the rate of improvement in health care delivery within these countries. Furthermore, the rate at which this increase has occurred exceeds the generational time of these countries, thereby eliminating a strictly genetic etiology. Consequently, environmental exposures have become the focus of research on the rising incidence of asthma and allergy as well as many other complex diseases. The National Institute of Environmental Health Sciences spearheads programs specifically to define and measure environmental exposures critical in human disease. The new Exposure Biology Program within the Genes and Environment Initiative of the National Institutes of Health targets gene and environmental exposure interactions resulting in human disease.

Development of complex diseases or disorders, including asthma, allergy, atherosclerosis, diabetes, and obesity, has been linked to multiple genes or quantitative trait loci within mammalian genomes (Casas et al. 2006; Shah et al. 2006). Multigene interactions are now suspected in most complex diseases (Chan et al. 2006; Motsinger et al. 2007; Yang et al. 2005). Increasingly, gene–environment interactions are also being examined for a role in the etiology of complex diseases (Colilla et al. 2003; Criswell et al. 2006; van Dellen et al. 2005; Williams et al. 2006). Through generalized fetal stress or specific biochemical reactions, *in utero* environmental exposures appear to mediate complex chronic diseases (cardiovascular disease, obesity, diabetes, asthma) having recognized genetic components (Genuis 2006; Martinez 2007; Okubo and Hogan 2004). Recent findings implicate *in utero* environmental exposures in developmental disorders such as autism (Ashwood et al. 2006). Although mild-to-moderate environmental exposures may not alter basic genetic information (DNA sequence), these exposures can determine the expression or repression of essential genes at developmentally critical points (Dolinoy et al. 2007; Li et al. 2003), thus contributing to chronic disease.

Altered lung function, increased asthma risk, and persistent lung function deficits in children (Gilliland et al. 2000, 2001; Li et al. 2000) have been associated with maternal smoking during pregnancy (*in utero* exposure). Environmental tobacco smoke (ETS) aggravates childhood asthmatic responses (Gilliland et al. 2000; Lindfors et al. 1999; Mannino et al. 2001), and premature adult cardiovascular disease in mice (Yang et al. 2004) is promoted by *in utero* ETS exposure. Altered lung function, exacerbation of symptoms, and acceleration of the disease processes seen with smoke exposure might arise from direct injury suffered by a developing fetus, by alteration of fetal gene expression, or through a combination of fetal injury and protective alteration of gene expression. Fetal sensitivity to ETS may be heightened, or ETS components may bioaccumulate as demonstrated by higher cotinine levels in neonates compared with their nonsmoking mothers who had received ETS exposure during pregnancy (Perera et al. 2004).

Ovalbumin (OVA) is an allergen commonly used in rodent models of allergic asthma. OVA sensitization by ip injection followed by inhalation challenge with OVA aerosol elicits expansion of the T-helper 2 (Th2) lymphocyte population. Production of Th2 cytokines follows, leading to airway hyperresponsiveness (AHR) and inflammation characterized by eosinophilia and appearance of OVA-specific immunoglobulin (Ig) E (Zhang et al. 1997). This sensitization and challenge protocol does not mimic the typical human experience of aerosol-only sensitization and challenge (Bice et al. 2000). However, aerosol-only OVA exposure of mice results in little or no OVA-specific serum IgE, and no eosinophilic inflammatory response.

Within this context, we designed experiments (Figure 1) to simulate the respiratory consequences to offspring of daily gestational exposures to ETS or filtered air (Penn et al. 2007). We combined daily *in utero* ETS exposure with postnatal OVA inhalation to test the hypothesis that *in utero* ETS exposure alters airway function and immune responses in adults. At 10 weeks of age, *in utero* ETS and *in utero* air mice received aerosol OVA exposures. The *in utero* ETS mice displayed significantly increased AHR without significant changes in histopathology, cytokine profiles, or antibody levels. At 15 weeks of age after OVA sensitization and challenge, *in utero* ETS mice not previously exposed to OVA exhibited significantly increased AHR compared with *in utero* air controls. ETS mice previously exposed to OVA demonstrated decreased numbers of bronchoalveolar lavage (BAL) eosinophils and polymorphonuclear leukocytes (PMNs), diminished AHR, and lower levels of interleukin (IL)-4, tumor necrosis factor (TNF)-α, and interferon (IFN)-γ compared with that of air controls.

In the present study we examined differential gene expression profiles in lungs of adult mice from four treatment groups [ETS-, saline-, and OVA-exposed (ESO); HEPA-filtered air-, S-, and O-exposed (ASO); E- and O-exposed, then O-exposed (EOO); A- and O-exposed, then O-exposed (AOO); Figure 1] using extracted mRNA. We extracted RNA from lung tissue taken concommitantly with histopathology, clinical pathology, and immunology samples (Penn et al. 2007). No sex differences were detected in immune responses, lung function, or histopathology of offspring in that study. Nevertheless, RNA for individual microarray analyses in this study was examined from females only (four females per treatment group) to eliminate any unrecognized sex effects. We used polymerase chain reaction (PCR) to further examine selected asthma-related and inflammatory genes in these subsets of each treatment group and to determine whether the differential gene expression levels were consistent with the pathophysiology and the immune system responses that we previously reported (Penn et al. 2007).

## Materials and Methods

### Animal protocols

We housed and handled 8-week-old BALB/c mice (Harlan, Indianapolis, IN) according to the *Guide for the Care and Use of Laboratory Animals* (Institute of Laboratory Animal Resources 1996). The Louisiana State University Institutional Animal Care and Use Committee approved all animal procedures. We handled all animals humanely and took care to ensure alleviation of pain and suffering.

### Exposures

**Figure 1:**
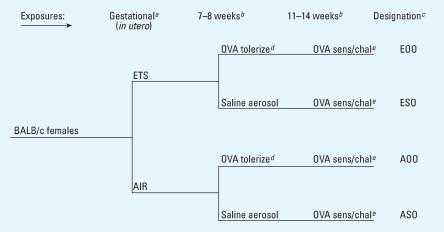
Exposures and timeline. Three 20-min inhalation exposures to 1% OVA in saline, every other day at 14 weeks plus an additional 20-min exposure 1 day before sacrifice at 15 weeks constituted OVA challenge; Abbreviations: AIR = HEPA-filtered air; ESO = ETS-, saline-, and OVA-exposed; ASO = HEPA-filtered air-, S-, and O-exposed; EOO = E- and O-exposed, then O-exposed; AOO = A- and O-exposed, then O-exposed. ***a***Days 1–19 of gestation. ***b***Weeks = age of offspring. ***c***Treatment group designation based on gestational, 8-week, and 11- to 14-week exposures. ***d***Tolerize = OVA tolerization established through inhalation of 1% OVA aerosol (in saline), 20 min daily for 10 days. ***e***Sens/chal = OVA sensitization was established by ip injections (80 μg OVA in 2.0 mg alum), one each at 11 and 13 weeks.

Pre- and postnatal exposure protocols have been described in detail (Penn et al. 2007). Briefly, we exposed pregnant mice in dynamic exposure chambers to either ETS mixed with HEPA-filtered air [final total suspended particle (TSP) concentration = 10 mg/m^3^; carbon monoxide concentration 44.5 ppm] or to HEPA-filtered air 5 hr/day for days 1–19 of gestation. Half of each of these groups inhaled 1% OVA aerosol (20 min/day for 10 days at 7–8 weeks of age) to produce tolerance, while the other half inhaled saline aerosol. We OVA-sensitized, challenged (Figure 1), and then sacrificed all mice 1 day after final challenge at 15 weeks of age to determine the effects of *in utero* ETS exposure on responses to OVA sensitization/challenge in mice not previously exposed to OVA (ESO, ASO) and on OVA tolerance in mice previously exposed to OVA (EOO, AOO). There were no postnatal offspring exposures to ETS after birth.

### Lung harvest and mRNA extraction

We excised left lung lobes [AOO (*n* = 21), EOO (*n* = 18), ASO (*n* = 20), ESO (*n* = 17)], placed them in RNAlater, and stored them at −80°C. Subsequently, we transferred these samples to 1 mL TRIzol (Invitrogen, Carlsbad, CA) and homogenized them using a Mixer Mill MM 300 (QIAGEN, Valencia, CA) with a copper bead. We added chloroform to the homogenate, mixed by inversion, and centrifuged at 4°C to separate phases. We transferred the RNA-containing aqueous phase to an RNeasy Micro Kit (QIAGEN). We followed the manufacturer’s protocol, omitting buffer RLT. We eliminated remaining DNA using a column RNase-Free DNase set (QIAGEN). We used an additional buffer RPE wash to remove residual salts, followed by an additional 2-min spin to evaporate residual ethanol.

We checked RNA samples quantity and purity with a NanoDrop ND-1000 Spectrophotometer (NanoDrop, Wilmington, DE). Values generated from the NanoDrop for all samples fell into the following ranges: 260/280 ratio: 2.09–2.17; 260/230 ratio: 2.20–2.27; concentration: 1,200–1,900 ng/μL. We performed further quality assays on 1:5 dilutions of RNA samples with an Agilent 2100 BioAnalyzer and Agilent RNA 6000 Nano Series II Kits (Agilent Technologies, Palo Alto, CA). All samples fell into the following ranges: 28S/18S ratio: 1.3–1.8, RNA integrity number: 8.5–9.6.

### Microarray assay

We assessed global gene expression in the lung in individual mice (four female mice per treatment group) on Affymetrix Mouse Genome 430 2.0 Arrays representing more than 39,000 transcripts with over 45,000 probe sets. The arrays were processed at the Research Core Facility of Louisiana State University Health Science Center-Shreveport.

Double-stranded cDNA synthesized from total RNA was used to create cRNA, which was then biotinylated, fragmented, and added to a hybridization cocktail that included probe array controls, bovine serum albumin, and herring sperm DNA. This cocktail was then hybridized (16 hr at 45°C) to oligonucleotide probes on a GeneChip Mouse 430 2.0 Array in a GeneChip Hybridization Oven 640. Immediately after hybridization, the array underwent an automated washing and staining protocol on a GeneChip Fluidics Station and was scanned with a GeneChip Scanner 3000. Data collection and processing of initial raw data were performed by a GeneChip Workstation. All gene chips and instrumentation were from Affymetrix (Santa Clara, CA).

### Gene expression analysis

We sent GeneChip Workstation data to Expression Analysis Systems (Durham, NC). Initially, a principal component analysis was used to determine clustering of experimental units. The analysis revealed clustering by treatment group with greater variation between groups than within groups (data not presented), thus validating subsequent comparisons between treatment groups. Before making these comparisons, data were subjected to reduction of invariant probes (REDI) to remove previously determined mal-performing probes from the data set. Pairs of treatment group data underwent permutation analysis for differential expression (PADE) that accounts for false positives by tabulating a false discovery rate (FDR) based on a permutation-generated reference curve (technical information on REDI and PADE analyses available at www.expressionanalysis.com). After REDI and PADE, expression data were tabulated for each remaining transcript, including individual transcript *p*-value, FDR, fold change, Affymetrix probe ID, gene symbol, and functional summary. All transcripts included in this study had a fold change of at least 1.5 (up or down), and both an individual *p*-value and FDR < 0.05.

### Pathway analyses

We analyzed gene expression data with the network- and pathway-building software Ingenuity Pathways Analysis 4.0; gene networks and canonical pathways were examined using the Ingenuity Analysis Knowledge Database (Ingenuity Systems, Redwood City, CA). We identified and scrutinized networks and pathways for phenotypic relevance. We identified select genes from the literature for confirmation by quantitative real-time PCR (qRT-PCR) analysis. We created custom networks to demonstrate the connections between the genes identified in our expression analyses.

### qRT-PCR

Cytokine changes that were apparent at the protein level [IL-4, IL-5, IL-10, IL-13, TNF-α (Penn et al. 2007)] were not seen in the filtered microarray data. In addition, several asthma-related genes did not pass all the filtering criteria and consequently were not identified in the filtered data as being differentially expressed, although preliminary data suggested otherwise. To clarify the status of these genes, the same RNA samples that had been subjected to the microarray assay underwent qRT-PCR to determine differential expression of 14 genes previously associated with asthma by other investigators (*Arg1*, *Ccl8*, *Ccl11*, *Ccl24*, *Ear11*, *Mcpt1*, *Sprr2a*, *Chi3l3*, *Chi3l4*, *Chia*, *Pde4b*, *Pde4d*, *Slc7a2*, *Tgfb1*), as well as 7 cytokine genes associated with asthma and/or pulmonary inflammation (*Ifn*γ, *Il1b*, *Il4*, *Il6*, *Il10*, *Il13*, *Tnfa*). The results of the four individuals in each treatment group were averaged, as they were with microarray analysis, to arrive at a fold change value for the treatment group.

We converted RNA from each sample to cDNA with the High Capacity cDNA Reverse Transcription Kit (Applied Biosystems, Foster City, CA). We performed real-time PCR on cDNA with a Model 7300 Real-Time PCR System with TaqMan Universal PCR Master Mix and TaqMan Gene Expression Assays (Applied Biosystems) for the selected genes. All data collected and analyzed here adhere to the guidelines for Minimal Information About a Microarray Experiment (MIAME 2007).

## Results

An ETS trend detected in pathophysiologic data (Penn et al. 2007) is supported in the present study by differential gene expression in ESO versus ASO mice. After REDI and PADE analyses, 80 transcripts in our study met all filtering criteria (fold change ≥ 1.5, FDR < 0.05, transcript *p*-value < 0.05). After removal of duplicate, unmapped, and poorly characterized transcripts, 60 unique transcripts (51 down-and 9 up-regulated) remained that were differentially expressed in the lungs of ESO versus ASO mice (Table 1). The actual limits of filtering criteria for these genes were fold change 3.25 to 1.67 and −3.37 to −1.58, FDR < 0.04, transcript *p*-value < 0.005. None of the asthma-related genes that we targeted for analysis (Table 2) were differentially expressed according to filtered microarray data in ESO versus ASO mice. However, when examined by qRT-PCR, four asthma-related genes were identified as up-regulated (*Arg1*, *Ccl24*, *Slc7a2*, *Mcpt1*). In each case, the fold change from the microarray analysis was very similar to that determined by qRT-PCR (Table 2). Neither microarray analysis nor qRT-PCR revealed differential expression of cytokine genes in ESO versus ASO mice (Table 3).

A significant ETS effect demonstrated pathophysiologically (Penn et al. 2007) is supported here by differential gene expression in EOO versus AOO mice. After REDI and PADE analyses, 85 transcripts were initially identified. Filtering criteria for these transcripts were: fold change 2.13 to 1.50 and −3.24 to −1.50, FDR ≤ 0.041, transcript *p*-value < 0.005. Removal of duplicate, unmapped, and poorly characterized transcripts left 31 down-regulated and 41 up-regulated transcripts differentially expressed in EOO mice relative to AOO controls (Table 4). Among the down-regulated genes, one asthma-related gene (*Ccl8*) met the filtering criteria. The qRT-PCR analysis of these samples verified results for *Ccl8* and indicated a down-regulation of 10 other asthma-related genes (*Arg1*, *Ccl11*, *Ccl24*, *Ear11*, *Mcpt1*, *Sprr2a*, *Chi3l3*, *Chi3l4*, *Chia*, *Slc7a2*) in EOO versus AOO mice (Table 2). Evaluation by qRT-PCR demonstrated down-regulation of 6 cytokine genes in EOO mice relative to AOO mice (*Il4*, *Il6*, *Il10*, *Il13*, *Tnfa*, *Il1b*; Table 4). Again, differential expression of these cytokines was not detected by microarray.

Gene expression comparisons of ASO versus AOO or ESO versus EOO mice reveal that differences exist primarily as a result of airway inflammation present in mice not previously exposed to OVA (ASO and ESO) and absent in mice previously exposed to OVA (AOO and EOO). AOO mice had 673 down- and 1,037 up-regulated transcripts relative to ASO mice, whereas EOO mice had 847 down- and 1,465 up-regulated transcripts relative to ESO mice (data available upon request). In each of these cases, unidentified, duplicate, and unmapped transcripts accounted for 15–25% of the total transcripts.

**Figure 2:**
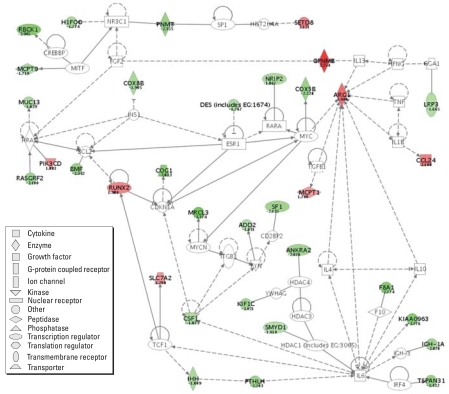
Literature-described gene network, including differentially expressed genes (ESO vs. ASO mice) from the lungs of *in utero* ETS-exposed mice versus *in utero* AIR-exposed mice that were subsequently OVA-sensitized and OVA-challenged (ESO, ASO). Green represents down-regulation and red up-regulation (fold-changes ≥ 1.5); white nodes represent fold-change < 1.5. Intensity of color increases with increasing fold change (numbers represent fold change values). Dotted lines indicate indirect relationships and solid lines direct interactions. All gene symbols are human homologue designations. Network constructed with Ingenuity Systems software.

For ESO versus ASO and EOO versus AOO comparisons, we organized genes into networks and identified genes within canonical pathways based on reference literature. The analyses allowed identification of experimentally denoted genes within defined functional pathways and networks based upon previously identified gene–gene or protein–protein relationships. Within the ESO–ASO comparison, 34 genes (8 up-regulated and 26 down-regulated) had demonstrable network connections (Figure 2). Among the down-regulated genes were 9 involved in promoting proliferation/differentiation/growth (*Tspan31*, *Pmnt*, *Pthlh*, *Sf1*, *Rasgrf2*, *Igh-V3609N*, *Ankra2*, *Csf1*, *Ihh*) and 4 associated with increased ubiquitination/apoptosis (*Mrcl3*, *Rbck1*, *Bmf*, *Lrp3*). Among the up-regulated genes were 3 associated with inhibition of proliferation (*Gpnmb*, *Setd8*, *Runx2*) and 1 promoting inflammatory and immune responses (*Pik3cd*), as well as 4 asthma-related genes (*Arg1*, *Ccl24*, *Slc7a2*, *Mcpt1*) identifiable only through qRT-PCR.

**Figure 3:**
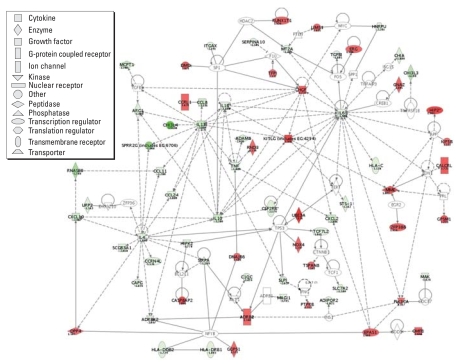
Literature-described gene network, including differentially expressed genes (EOO vs. AOO mice) from the lungs of *in utero* ETS-exposed mice versus *in utero* AIR-exposed mice, that were OVA-tolerized and then OVA-sensitized and challenged (EOO, AOO). Green represents down-regulation and red up-regulation (fold-changes ≥ 1.5); white nodes represent fold change < 1.5. Intensity of color increases with increasing fold change (numbers represent fold change values). Dotted lines indicate indirect relationships and solid lines direct interactions. All gene symbols are human homologue designations. Network was constructed with Ingenuity Systems software.

Within the EOO–AOO comparison, 71 genes (43 down-regulated and 28 up-regulated) had documented network connections (Figure 3), including the 14 genes identified by qRT-PCR. Of 43 down-regulated genes, 32 enhance immune and inflammatory responses or have been implicated in asthma pathogenesis, including 6 cytokine (*Il1b*, *Il4*, *Il6*, *Il10*, *Il13*, *Tnfa*), 4 chemokine (*Ccl8*, *Ccl11*, *Ccl24*, *Cxcl10*), and 8 asthma-related genes (*Arg1*, *Ear11*, *Mcpt1*, *Sprr2a*, *Chi3l3*, *Chi3l4*, *Chia*, *Slc7a2*). Among the up-regulated genes were 6 involved in positive regulation of proliferation/differentiation/growth (*Kitlg*, *Runxlt1*, *Gnaz*, *Pik3ca*, *Ptprb*, *Tmrsf3*), 7 associated with increased ubiquitination/apoptosis (*Ube3a*, *Hip2*, *Casp8ap2*, *Gmfb*, *Erg*, *Pja2*, *Usp53*), and 4 involved in suppression of immune responses (*Hgf*, *Ppapdc2*, *Rnd3*, *Dpp4*). The majority of differentially expressed genes that we discovered affecting immune function are not discussed within this report but are discussed in the Supplementary Material [Supplementary Table 1 (http://www.ehponline.org/members/2007/10358/suppl.pdf)].

## Discussion

This is the first report to identify gene expression changes in the adult lung following ETS exposure *in utero*. In addition the gene expression results presented here (microarray and qRT-PCR) provide a molecular framework within which to consider the pathophysiologic, lung function, and inflammatory responses we recently reported (Penn et al. 2007).

The ETS exposure level (TSP = 10 mg/mm^3^) used in this study is the same used in our other *in utero* (Yang et al. 2004) and earlier adult exposure studies (Bowles et al. 2005; Penn and Snyder 1993). The associated steady-state CO levels are approximately double those detected in a typical indoor smoking area (Penn et al. 1994). This exposure level, while higher than that found in a smoking household, is well below that used in numerous published studies. In light of the indirect (*in utero*) nature of these exposures and the lack of literature on the delivered fetal dose with varying levels of maternal ETS exposure, we selected an exposure that we felt would be sufficient to elicit a detectable response while not being beyond physiologic reality.

We have carried out multiple *in utero* ETS exposure experiments with 13–30 mice per mixed-sex treatment group (Penn et al. 2007; Rouse RL and Penn AL, unpublished observations). In these studies, there were no demonstrable sex-related differences among offspring in immune responses, histopathology, lung function, or clinical pathology. These results do not predict a significant sex difference in gene expression. Nevertheless, only female offspring samples from our earlier study (Penn et al. 2007) were used in the present gene studies to rigorously control for subtle, undetected sex influences. The gene expression results we report here for those female offspring correlate well with the pathology and lung function results from that earlier study.

Another recent *in utero* study demonstrated differential DNA methylation leading to modulated gene expression as a result of altered maternal diet (Dolinoy et al. 2006). No sex differences were detected within that study as well. Sex differences have, however, been reported for some adult responses to *in utero* stresses. In an earlier collaborative study, we demonstrated that *in utero* ETS exposure (identical to that used in this experiment) produces significant acceleration of atherosclerotic plaque development in male apoE^−/−^ mice (Yang et al. 2004). A similar trend existed within female mice, but it did not reach a level of statistical significance. In contrast, there is a report that male but not female BALB/c mice that had been exposed *in utero* to mainstream smoke from 1R3F research cigarettes [not ETS from the lower tar, lower nicotine 1R4F cigarettes (Davis et al., 1984) used here] displayed increased tumor incidence and size associated with decreased cytotoxic T-lymphocyte activity after injection of EL4 lymphoma cells (Ng et al. 2006). The variation in these reports on sex effect illustrates the difficulty in predicting adult sex-specific responses associated with prior *in utero* stresses and strongly suggests that different molecular mechanisms operate dependent on test agent, exposure conditions, subject strain, and experimental end points.

In ESO versus ASO mice, we identified differentially expressed genes that participate primarily in cell proliferation, cell motility/elasticity, cell survival, and general cell metabolism (Table 1). These biological functions are mediated at the molecular level by down-regulation of adhesion/apoptosis signaling molecules, transcription activating DNA-binding motifs, adenosine triphosphate/guanosine triphosphate (ATP/GTP) metabolic reagents, and cytoskeletal structural components and binding elements. Up-regulated genes contribute to these biological processes through negative regulation of cell proliferation, repression of transcription, and chromatin remodeling. Again, this is accomplished through altered adhesion, transcription, metabolism, and cytoskeletal elements

A single kinase gene (*Pik3cd*) known to participate in immune and inflammatory signaling through nuclear factor kappa B (NfκB), extracellular signal-regulated kinase (Erk), and mitogen-activated protein kinase (Mapk) pathways was up-regulated. Nfκb, Erk, and Mapk participate in inflammation processes (Roux and Blenis 2004), including those in the lung (Wuyts et al. 2003). Pik3cd also has been linked to the increased vascular permeability observed in inflammation (Lee et al. 2006). Four genes linked to asthma or lung inflammation (*Arg1*, *Ccl24*, *Slc7a2*, *Mcpt1*) that did not pass all the microarray filtering criteria were confirmed by qRT-PCR as being up-regulated in ESO versus ASO mice, which is consistent with an enhanced lung response in ETS-exposed mice. Both Arg1 and Slc7a2 have been implicated in arginine metabolism (Zimmermann et al. 2003). Arginine serves as a substrate for both arginase 1 and nitric oxide (NO) synthetase. Thus, increases in arginine metabolism catalyzed by arginase 1 are believed to decrease the production of NO via NO synthetase. Normal relaxation of smooth muscle is NO dependent, and decreases in NO are thought to contribute to smooth muscle–mediated broncho-constriction, such as that seen in asthma. Scl7a2 (CAT2) is a cationic amino acid transporter involved in arginine transport. Altered arginine transport affects the arginine metabolic pathway and may also alter NO production. Ccl24 (eotaxin 2) plays a role in eosinophil recruitment to the lung in allergic asthma (Panina-Bordignon and D’Ambrosio 2003). The Mpct family contains mast cell-specific proteases that participate in mast cell activation and degranulation (Small-Howard and Turner 2005). Changes in cytoskeletal elements and binding can confer relaxed cell-to-cell attachment and facilitate infiltration of inflammatory elements. The gene expression profile reported here indicates that adult mice exposed *in utero* to ETS have increased lung inflammation in response to OVA relative to *in utero* air-exposed cohorts. These results support the functional, histopathologic, and immune system changes we have documented in OVA-sensitized and challenged mice (Penn et al. 2007).

Figure 2 illustrates a literature-based gene network to which our differentially regulated gene findings (ESO vs. ASO comparison) have been applied. This network brings together, through our data, groups of relationships that become associated for the first time, creating a custom network of differential gene response for our experiment. This custom network shows that the differences seen between our treatment groups are based on differential regulation of a dispersed and varied group of genes in clustered relationships with inflammatory/asthma genes that are not differentially regulated. The majority of these differentially regulated genes does not have direct ties to the immune system or previously defined inflammatory responses. None of the well-defined asthma/inflammatory genes (*Il1b*, *Il4*, *Il10*, *Il13*, *Il6*, *Tnfa*, *Infg*, *Tgfb1*) are differentially expressed, although they connect many of the genes that are. This is not surprising, as the comparison is between two groups (ESO, ASO) that both exhibit marked eosinophilic airway inflammation following classic OVA sensitization/challenge. However, the few asthma/inflammatory genes that are differentially expressed (*Pik3cd*, *Arg1*, *Slc7a2*, *Ccl24*, *Mcpt1*) are all up-regulated in ETS-exposed mice. The mild enhancement of inflammatory/immune response indicated by these gene changes is reflected in the pathophysiologic responses of the two groups (Penn et al. 2007) and raises concern that ETS may mediate increased inflammatory injury or alter immune response upon initial exposure to an infectious agent such as respiratory syncytial virus (Phaybouth et al. 2006).

In EOO versus AOO mice, differentially expressed genes participate in adaptive immune responses, cell proliferation, and survival, and cell cycle control (Table 4). These processes are controlled through down-regulated adhesion, major histocompatibility complex (MHC), immune, and proinflammatory molecule genes, including cytokines and chemokines. Up-regulated genes influence these processes through alterations in G-protein receptor signaling and cell proliferation, differentiation, metabolism, and morphology. Consistent down-regulation of critical inflammatory genes in ETS- and doubly OVA-exposed mice was confirmed through qRT-PCR, revealing down-regulation of 17 asthma-related and/or inflammatory genes (*Arg1*, *Ccl8*, *Ccl11*, *Ccl24*, *Ear11*, *Mcpt1*, *Sprr2a*, *Chi3l3*, *Chi3l4*, *Chia*, *Slc7a2*, *Il4*, *Il6*, *Il10*, *Il13*, *Il1b*, *Tnfa*).

Genes directly or indirectly inhibiting immune responses were up-regulated. Genes promoting immune responses were down-regulated, including genes for the proinflammatory/Th2 cytokines IL-1b, IL-4, IL-6, IL-10, IL-13, and TNF-α. Proinflammatory cytokine genes (*Il1b*, *Il6*, *Tnfa*) were down-regulated in EOO mice, decreasing innate inflammatory potential, as seen in endotoxin tolerance (Cook 1998). Down-regulation of Th2 cytokine genes (*Il4*, *Il10*, *Il13*), as described in our data, has been reported in alloreactive T cells in which tolerance has been induced and immune rejection suppressed (Taylor et al. 2002). The gene for IL-10, which is essential for the eosinophilic inflammation seen in asthma (and in this mouse model), is down-regulated, resulting in decreases in mucus production and eosinophilic inflammation without decreased IgE or IL-4 (Yang et al. 2000).

Almost all differentially expressed genes participating in the immune, inflammatory, and asthma reactions are modulated toward reduced responsiveness in the *in utero* ETS-exposed mice (EOO vs. AOO). The effect of this differential expression—decreased antigen-presenting capacity and dampened immune signaling—implies decreased innate and adaptive immune responses to new challenges, as well as reduced inflammation. We have previously demonstrated these dampened responses in OVA-tolerized mice that were subsequently sensitized and challenged (Penn et al. 2007).

Figure 3 illustrates a literature-based network of relationships between differentially expressed genes that we identified in the EOO versus AOO comparison. This network connects, through our data, groups of previously described relationships that have not been previously associated. The resulting custom network demonstrates that the significant, observed, immune response differences seen in this comparison (EOO vs. AOO) are related to differential regulation of genes that collectively suppress immune responsiveness. Major inflammatory/immune genes (*Il1b*, *Il4*, *Il10*, *Il13*, *Il6*, *Tnfa*) that are down-regulated in EOO mice relative to AOO mice appear connecting many up- and down-regulated genes negatively regulating the immune response. The specificity of this suppressive modulation is of concern. Would exposure to alternate antigens within this environment of immune down-regulation elicit appropriate innate and/or adaptive responses? Agents, identified by MHC molecules or neutralized by effector cells that are down-regulated by *in utero* ETS exposure, could escape detection or elicit suboptimal immune responses.

Although our main interest was the effect of ETS on lung responses to OVA in both sensitized and tolerized mice, it is noteworthy that the number and magnitude of differences between ESO and EOO mice greatly exceed those between ASO and AOO mice (Tables 1 and 2). In all the asthma-related genes examined, ESO–EOO differences were consistently greater (qRT-PCR) than ASO–AOO differences. This net effect is a result of the mild-to-moderate increase in responsiveness of ESO mice compared with ASO mice and the much greater suppression of response in EOO mice relative to AOO mice. The relative responsiveness or reactivity of these four groups of mice, indicated by their general gene expression profiles as well as by their relative expression of specific asthma/inflammatory genes, is supported by histopathology, BAL cell counts and cytokine analyses, immunoglobulin levels, and AHR (Penn et al. 2007). In each comparison, additional candidate genes (white nodes in Figures 2 and 3) warrant future investigation.

In our comparison of gene expression between ESO mice and ASO mice, it was evident that genes for cell proliferation, growth, and general metabolic processes were more suppressed in the most reactive group (ESO). Inflammation is associated with proliferation of innate and adaptive immune cells. However, activation of the p38 pathway (Diehl et al. 2000) and Pik3cd (Donahue and Fruman 2004) can result in cell cycle arrest and halt cell proliferation and growth in lymphocytes. Findings in the EOO versus AOO mice indirectly support the findings in ESO versus ASO mice. The least-responsive group (EOO) had a differential expression profile that was indicative of increased cell proliferation, growth, and metabolism. Because our lung samples represent total lung RNA, proliferation status of nonimmune cells is also reflected in the differential expression. Fibroblasts have been implicated in maintenance of the local environment (extracellular matrix) within tissues, including down-regulation of inflammatory responses (Buckley et al. 2001). Proliferation of regulatory fibroblasts might overshadow down-regulation of proliferation in immune cells.

The fate of T cells, activation or anergy, has been tied to both cell cycle progression (Wells et al. 2000) and intracellular calcium flux (Gavin et al. 2002). Evidence for regulation of both the cell cycle and of ion channels is presented in our data (Tables 1 and 4). The ultimate fate of the immune response will depend on the character of the T cells involved (T-effector vs. T-regulatory) as well as on the presence of co-stimulators, MHC expression, and innate immune elements. An attenuation of innate and adaptive responses, if nonspecific, might threaten the ability of the least-responsive group (EOO) to respond appropriately to an infectious agent. Similarly, excessive airway inflammation might impair defenses or increase injury during infection (Beisswenger et al. 2006) in the most-responsive group (ESO). Thus, either extreme of the ETS-based responsive dichotomy may be undesirable (Clark and Kupper 2005).

Our data comparing two aerosoltolerized groups (EOO, AOO) illuminate the complexity of immune tolerance. The dynamic nature of tolerance, involving the down-regulation and inhibition of numerous biological processes that collectively potentiate the immune system, is well illustrated by our data. Although anergy of specific T-cell lines plays a significant role in tolerance, many other “players” (dendritic cells, PMN’s, mast cells, macrophages, eosinophils) are suppressed via multiple mechanisms, including structural and enzymatic changes yielding functional impairments. *In vivo*, tolerance is the sum of suppressed stimulation/activation (decreased antigen presentation, T-cell anergy, dampened intracellular signaling) and impaired effector function (depressed chemotaxis, protease inhibition, suppressed cell mobility). Our data define multiple sites and diversities in mechanisms involved in inhibiting the immune system and achieving tolerance.

In the present study we also demonstrate that varying degrees of responsiveness can exist within the tolerant population. Although both EOO and AOO mice demonstrated no IgE or eosinophilic inflammation (and thus were considered tolerant), the AOO group had higher levels of Th2 cytokines, BAL inflammatory cells, airway hyperresponsiveness, and airway pathology (Penn et al. 2007). In all cases, AOO mice demonstrated more response in pathology, cytokine production, immune cell accumulation in the airway, and airway hyperreactivity, and in many of these cases the differences were significant. Differential gene expression data reveal more gene alterations inhibiting the immune response in EOO versus AOO mice. These differences, initiated by an environmental exposure limited to the gestational period, may not be totally antigen specific and therefore are potentially detrimental. Certainly, the difference between responsiveness in EOO and AOO mice is a reflection of the mechanisms involved in tolerance induction.

Alteration of gene expression mediated by *in utero* environmental exposure represents a change in phenotype determined by gene-environment interaction. The mechanism by which these gene expression changes are orchestrated is not yet defined, but epigenetic involvement is likely (Drake et al. 2005; Rahnama et al. 2006; Waterland and Jirtle 2003). Given the number and variety of genes that we found to be differentially regulated, along with the indirect and relatively mild nature of exposures that culminated in demonstrable response differences in immunology, histopathology, clinical pathology, and lung function, the search for single candidate genes may need to evolve to an examination of global epigenetic alterations. In most cases, gene–environment interactions may cause transient, reversible, or noncritical alterations in gene expression, or no detectable change in phenotype. However, the ability of relatively mild *in utero* environmental exposures during embryonic development to modulate adult gene expression may move chronic adult diseases (atherosclerosis, obesity, diabetes, allergy, asthma) into the realm of early developmental disorders such as childhood diabetes, leukemia, and autism that also appear to be environmentally modulated.

Genes recognized in this study are present in other tissues and may have varied and diverse functions beyond those described here. However, the functions delineated within this present study are consistent with the pathologic and physiologic differences defined by the study exposures. Gene ontogeny has been examined in context of the complexity of an *in vivo* system. Further refinement of specific cell types involved and specific mechanisms of differential gene regulation are ongoing, particularly gene-specific and global changes in epigenetic modifications.

## Conclusion

*In utero* ETS exposure alters gene expression in the lung of adult BALB/c mice in response to OVA exposure, regardless of whether that exposure was sensitizing or tolerizing. The results support a gene–environment interaction that results from *in utero* ETS exposure and that alters the phenotype of adult mice as defined by their gene expression and inflammatory responses to an allergen (OVA). The strength of these findings is re-enforced by the consistency of relative group responses across gene expression data, airway function (reactivity) changes, presence of airway inflammatory mediators (cytokines, cells), and lung histopathology. Our data implicate milder, widespread gene expression changes rather than (or in addition to) larger more discrete alterations in single gene expression as the mechanism through which these *in utero* exposures alter adult lung responses.

## Figures and Tables

**Table 1:** Genes differentially expressed by microarray in OVA-sensitized mice exposed *in utero* to ETS or AIR (ESO vs. ASO).^a^

Affymetrix ID	Fold change	FDR	Transcript *p*-value	Gene symbol	Protein family	Function	Process
1448303_at	3.25	0.040	0.0018	*Gpnmb*	Enzyme	Integrin binding	Inhibit proliferation
1441557_at	2.12	0.040	0.0008	*Setd8*	Methyltransferase	Histone (H4) methylation	Control cytokinesis
1424706_at	2.02	0.037	0.0003	*Zfp51*	Zinc finger protein	DNA/protein binding	Unknown
1444396_at	2.01	0.040	0.0008	*Trp53inp2*	Unknown	Unknown	Unknown
1424704_at	1.98	0.037	0.0002	*Runx2*	Transcription regulator	Transcription inhibitor	Inhibit proliferation
1421525_a_at	1.92	0.037	0.0002	*Birc1e*	Receptor (PRR)	Unknown	Macrophage activation/death
1450541_at	1.89	0.037	0.0003	*Pvt1*	Unknown	Unknown	Unknown
1453281_at	1.88	0.040	0.0006	*Pik3cd*	Kinase	VEGF activation	Promote immune responses
1434500_at	1.67	0.037	< 0.0001	*Ttyh2*	Ion channel protein	Chloride channel	Chloride channel regulation
1456868_at	−3.37	< 0.001	0.0004	*Mrcl3*	Myosin light chain	Cytoskeleton actin binding	Motility, elasticity, apoptosis
1457610_at	−2.91	< 0.001	0.0006	*Rbck1*	Transcription regulator	Zinc and protein binding	Ubiquitinization
1433774_x_at	−2.81	< 0.001	0.0006	*Cog1*	Tranport protein	Protein binding	Golgi transport
1441840_x_at	−2.78	< 0.001	0.0001	*Kiaa0963*	Unknown	Unknown	Unknown
1447530_at	−2.77	< 0.001	0.0012	*F8a*	F actin binder	Endosomal binding/motility	Endosomal formation/function
1440180_x_at	−2.76	< 0.001	< 0.0001	*Zbtb3*	Zinc finger domain	DNA/protein binding	Unknown
1458354_x_at	−2.62	< 0.001	0.0006	*Krt28*	Keratin	Unknown	Unknown
1443829_x_at	−2.55	< 0.001	0.0001	*Coasy*	Kinase	Adenylyltransferase	Coenzyme A regulation
1455853_x_at	−2.43	< 0.001	0.0009	*Tspan31*	Tetraspanin	Transmembrane protein	Proliferation, differentiation, growth
1449804_at	−2.36	0.040	0.0036	*Pnmt*	Methyltransferase	Tyrosine metabolism	Growth
1458325_x_at	−2.34	0.040	0.0043	*Bmf*	Protein binding	Cytoskeleton myocin binding	Cell repair/induce apoptosis
1436149_at	−2.23	0.037	0.0020	*Cox5b*	Enzyme	Cytochrome c subunit	Oxidative phosphorylation
1427527_a_at	−2.20	< 0.001	0.0001	*Pthlh*	Hormone	Calcium binding	Proliferation, differentiation, growth
1443752_at	−2.17	< 0.001	0.0009	*Kiaa1529*	Unknown	Unknown	Unknown
1438638_x_at	−2.17	0.037	0.0020	*Fam116b*	Unknown	Unknown	Unknown
1459766_x_at	−2.14	< 0.001	0.0004	*Sf1*	Transcription regulator	Activate transcription	Proliferation, differentiation, growth
1421788_x_at	−2.13	< 0.001	0.0011	*Klk13*	Peptidase	Unknown	Unknown
1421621_at	−2.10	< 0.001	0.0003	*Rasgrf2*	Guanyl exchange factor	Guanyl nucleotide exchange	Growth and signaling
1452538_at	−2.08	< 0.001	0.0006	*Igh-V3609N*^b^	Ig super-family	Antigen binding	Proliferation, differentiation, growth
1456746_a_at	−2.08	< 0.001	0.0005	*Cd99l2*	Membrane protein	Unknown	Unknown
1441935_at	−2.07	< 0.001	0.0002	*Ankra2*	Transcription regulator	Activate transcription	Proliferation, differentiation, growth
1419216_at	−2.04	0.040	0.0032	*Azi1*	Centrosomal protein	Unknown	Unknown
1426325_at	−2.02	0.037	0.0015	*Kif1c*	Kinesin	Cytoskeleton binding protein	Motility and Golgi transport
1424845_a_at	−1.94	0.037	0.0011	*Cep68*	Centrosomal protein	Unknown	Unknown
1449218_at	−1.91	0.037	0.0019	*Cox8b*	Enzyme	Cytochrome assembly	Oxidative phosphorylation
1425155_x_at	−1.88	0.037	0.0017	*Csf1*	Transcription regulator	Activate transcription	Proliferation, differentiation, growth
1433792_at	−1.87	0.040	0.0026	*Nrip2*	Transcription regulator	Inhibit transcription	Unknown
1439745_at	−1.84	0.037	0.0010	*Cacng7*	Ion channel protein	Calcium channel	Calcium channel regulation
1425120_x_at	−1.83	0.040	0.0019	*Fam14a*	Unknown	Unknown	Unknown
1454634_at	−1.83	0.037	0.0014	*Fuk*	Kinase	Glycoprotein phosphorylation	Leukocyte trafficking
1419387_s_at	−1.83	< 0.001	0.0005	*Muc13*	Mucin	Transmembrane protein	Unknown
1440368_at	−1.83	0.040	0.0023	*Jmjd2b*	DNA binding	Unknown	Unknown
1459977_x_at	−1.81	0.037	0.0011	*Cox10*	Enzyme	Heme synthesis	Oxidative phosphorylation
1450704_at	−1.81	0.037	0.0010	*Ihh*	Enzyme	Transferase	Proliferation, differentiation, growth
1416518_at	−1.77	0.040	0.0019	*H1foo*	H1 histone protein	DNA and chromatin binding	Chromatin folding
1417305_at	−1.77	0.040	0.0019	*Des*	Desmin	Cytoskeleton binding protein	Smooth muscle motility/elasticity
1435015_at	−1.75	< 0.001	0.0005	*Zfp787*	Zinc finger protein	DNA/protein binding	Unknown
1426274_at	−1.74	< 0.001	0.0001	*Slc9a8*	Solute carrier protein	Sodium/hydrogen exchange	Unknown
1439144_at	−1.74	< 0.001	0.0001	*Cwf19l1*	Protein binding	Unknown	Cell cycle control
1430236_s_at	−1.74	< 0.001	0.0001	*Gdsm2*	Unknown	Unknown	Unknown
1450538_s_at	−1.72	< 0.001	0.0003	*Mcpt9*	Peptidase	Peptide catabolism	Mast cell degranulation
1448927_at	−1.70	0.040	0.0012	*Kcnn2*	Ion channel protein	Potassium channel	Potassium channel regulation
1427409_at	−1.68	0.037	0.0010	*9-Mar*	Ring finger protein	Unknown	Unknown
1436083_at	−1.67	0.037	0.0009	*Lrp3*	Receptor (TM)	Lipoprotein binding	Adhesion, apoptosis
1434463_at	−1.65	0.037	0.0009	*Bfsp2*	Phakinin	Cytoskeleton component	Motility, elasticity, structure
1459073_x_at	−1.63	0.040	0.0011	*Fgf14*	Growth factor	Ion channel protein binding	Sodium channel regulation
1421975_a_at	−1.63	0.040	0.0015	*Add2*	Adducin	Cytoskeleton actin binding	Motility, elasticity, structure
1421329_a_at	−1.61	0.037	0.0004	*Smyd1*	Transcription regulator	Inhibit transcription	Chromatin remodeling
1426493_a_at	−1.61	< 0.001	0.0001	*Kifc2*	Kinesin	Cytoskeleton binding protein	Motility and Golgi transport
1432883_at	−1.59	0.037	0.0006	*Wdr87*	Unknown	Unknown	Unknown
1420569_at	−1.58	< 0.001	< 0.0001	*Chad*	Chondroadherin	Unknown	Chrondrocyte adhesion

AIR, HEPA-filtered air. Gene annotations are from Affymetrix GeneChip Mouse Genome 430 2.0 Array (http://www.affymetrix.com/products/arrays/specific/mouse430_2.affx).

a Mice were exposed *in utero* to ETS or AIR (HEPA-filtered air), and then OVA-sensitized by ip injections and challenged with aerosol OVA as described in “Material and Methods.”

b Human homologue IGH-1A.

**Table 2:** Differential expression of asthma-related genes: microarray versus qRT-PCR.

	*Sprr2a*	*Ccl8*	*Ccl11*	*Ccl24*	*Ear11*	*Mcpt1*	*Arg1*	*Chi3l3*	*Chi3l4*	*Chia*	*Pde4b*	*Pde4d*	*Slc7a2*	*Tgf* β*1*
AOO vs ASO							
Array	−5.6^a^	−1.8	−3.8^b^	−2.6^a^	−4.6^b^	−1.3^a^	^−^6.3^a^	−1.5^d^	−12.6	−2.2^a^	1.6^b^	1.1^d^	−1.3^d^	−1.1^a^
PCR	−17.3	−2.9	−3.6	−4.7	−5.0	−2.0	−5.8	−3.6	−18.5	−3.9	1.3	1.1	−1.6	−1.1
ESO vs ASO							
Array	1.1^a^	−1.1^a^	1.3^a^	1.8^a^	1.3^a^	1.2^a^	2.2^a^	1.1^d^	−1.0^a^	1.2^a^	−1.1^d^	1.1^d^	1.7^b^	−1.1^a^
PCR	−1.0	1.0	1.2	2.1	1.4	1.7	2.4	1.2	−1.1	1.2	1.1	1.0	1.7	1.2
EOO vs ESO							
Array	−7.3^c^	−5.2	−7.0	−5.2^a^	−23.8	−1.5	−22.0	−3.6^e^	−45.7	−4.3	4.8	1.1^d^	−1.6^b^	−1.6^a^
PCR	−131.4	−8.2	−7.7	−17.0	−41.3	−11.2	−26.8	−17.6	−228.5	−8.3	1.0	1.1	−4.0	−1.8
EOO vs AOO							
Array	−1.4^d^	−3.2	−1.5^a^	−1.1^a^	−4.1^a^	1.0^a^	−1.6^a^	−2.1^e^	−3.7^a^	−1.6^a^	−1.1^d^	1.1^d^	−1.2^d^	−1.3^a^
PCR	−7.9	−2.8	−1.7	−1.8	−5.7	−3.2	−1.9	−4.1	−13.6	−1.8	−1.1	1.1	−1.5	−1.4

a Did not pass transcript *p*-value or FDR.

b Did not pass FDR.

c Average of two transcripts for same gene; one did not pass FDR.

d Average of two transcripts; did not pass transcript *p*-value or did not pass FDR.

e Average of two transcripts for same gene. All values represent fold difference (in both magnitude and direction) between the group named first and the group listed second.

**Table 3:** Differential expression of cytokine genes: mircroarray versus qRT-PCR.

	*Ifn*γ	*Il1b*	*Il4*	*Il6*	*Il10*	*Il13*	*Tnf*α
AOO vs ASO														
Array	−1.1^a^	−1.1^a^	−1.2^b^	−1.0^a^	−1.1^a^	−1.4^b^	^−^1.1^a^
PCR	−1.1	−1.1	−2.1	−1.6	−1.7	−4.9	−1.2
ESO vs ASO														
Array	−1.0^a^	−1.4^a^	1.3^a^	−1.1^a^	−1.1^a^	−1.3^a^	−1.1^a^
PCR	1.1	−1.1	1.3	−1.3	−1.2	−1.1	1.0
EOO vs ESO														
Array	−1.0^a^	−1.3^a^	−1.7^c^	−1.0^a^	−1.2^a^	−1.1^a^	−1.2^a^
PCR	−1.6	−1.6	−4.7	−2.2	−4.8	−17.2	−2.1
EOO vs AOO														
Array	−1.0^a^	−1.6^a^	−1.1^a^	−1.0^a^	−1.0^a^	−1.0^a^	−1.1^a^
PCR	−1.3	−1.6	−1.8	−1.9	−3.2	−3.8	−1.8

a Did not pass transcript *p*-value or FDR.

b Average of two transcripts, did not pass transcript *p*-value or FDR.

c Did not pass FDR. All values represent fold difference (in both magnitude and direction) between the group named first and the group listed second.

**Table 4:** Genes differentially expressed by microarray in OVA-tolerized mice exposed *in utero* to ETS or AIR (EOO vs. AOO).^a^

Affymetrix ID	Fold change	FDR	Transcript *p*-Value	Gene symbol	Protein family	Function	Process
1425206_a_at	2.13	< .001	0.0002	*Ube3a*	Enzyme	Ubiquitin conjugation	Protein catabolism
1415854_at	1.94	< .001	0.0006	*Kitlg*	Cytokine	Erk, Mapk, Akt activation	Proliferation signaling
1417188_s_at	1.94	< .001	0.0009	*Hip2*	Transcription regulator	Activate transcription	Ubiquitin regulation
1449217_at	1.87	0.041	0.0028	*Casp8ap2*	Protease activator	NFκB activation	Apoptosis/survival signaling
1450924_at	1.83	< .001	0.0005	*Hdgfrp3*	Protein binder	Bind growth factor	Regulation of growth factor
1422975_at	1.80	< .001	< .0001	*Mme*	Peptidase	Degrade bradykinin/elastin	Extracellular matrix repair
1428025_s_at	1.80	< .001	0.0004	*Pitpnc1*	Transport protein	Phosphatidylinositol binding	Phosphatidylinositol regulation
1436917_s_at	1.76	0.041	0.0031	*Gpsm1*	G-protein receptor	Activate GTPases	Regulate G-protein receptor activity
1421230_a_at	1.74	0.041	0.0033	*Msi2h*	Unknown	Unknown	Unknown
1418489_a_at	1.73	< .001	0.0001	*Calcrl*	G-protein receptor	Mobilize calcium/cAMP	Control smooth muscle migration
1451866_a_at	1.73	< .001	0.0003	*Hgf*	Cytokine	Erk, Mapk activation	Suppress dendritic cell activation
1453139_at	1.72	< .001	0.0002	*Nudt12*	Phosphatase	Hydrolase	Nucleotide regulation
1425845_a_at	1.68	0.041	0.0012	*Shoc2*	Protein binder	Co-inhibitor of Raf1	Regulate Ras pathway
1447926_at	1.67	0.041	0.002	*Arl5*	GTPase	Unknown	Unknown
1417069_a_at	1.67	0.041	0.0015	*Gmfb*	Cytokine	NFκB activation	Apoptosis/survival signaling
1448665_at	1.67	0.041	0.0015	*Dmd*	Protein binder	Actin binding	Cytoskeletal anchoring
1451146_at	1.66	0.041	0.0016	*Zfp386*	Transcription regulator	Chromatin/DNA binding	Unknown
1449888_at	1.66	0.041	0.0016	*Epas1*	Transcription regulator	Activate Vegf	Control vascular remodeling
1418231_at	1.64	0.041	0.0011	*Lims1*	Adapter protein	Pi3k, Akt activation	Integrin signaling
1420514_at	1.64	0.041	0.0016	*Tmem47*	Unknown	Unknown	Unknown
1437784_at	1.63	< .001	0.0002	*Runx1t1*	Transcription regulator	Increase Myc, Jun expression	Proliferation/growth
1437668_at	1.63	0.041	0.0014	*Ccrl1*	G-protein receptor	Activate T-cell/dendritic cytokines	Immune signaling
1426517_at	1.61	< .001	0.0002	*Gnaz*	Enzyme	GTP/Erk, Mapk activation	Differentiation signaling
1447944_at	1.61	< .001	0.0001	*Zkscan1*	Unknown	Unknown	Unknown
1425370_a_at	1.60	0.041	0.0021	*Erg*	Transcription regulator	Increase Tgfb2 expression	Inhibit apoptosis
1451827_a_at	1.60	0.041	0.0011	*Nox4*	Enzyme	NADPH oxidase	ROS metabolism/inhibit proliferation
1428345_at	1.58	0.041	0.0018	*Ppapdc2*	Phosphatase	Diphosphate phosphotase	Inhibit PMN-mediated inflammation
1429776_a_at	1.57	0.041	0.0022	*Dnajb6*	Small heat shock protein	Co-chaperone (hsp70)	Protein transport and folding
1419805_s_at	1.57	0.041	0.0021	*Ggps1*	Enzyme	Prenyltransferase	Sterol synthesis
1437302_at	1.56	0.041	0.001	*Adrb2*	G-protein receptor	Erk, Mapk activation	Motility/adhesion signaling
1418780_at	1.55	< .001	0.0001	*Cyp39a1*	Enzyme	Hydroxylase	Lipid metabolism
1437982_x_at	1.54	0.041	0.0015	*Cox15*	Enzyme	Cytochrome c assembly	Oxidative phosphorylation
1438530_at	1.54	< .001	0.0002	*Tfpi*	Protease inhibitor	Inhibition of F10	Coagulation regulation
1416701_at	1.53	0.041	0.0016	*Rnd3*	Enzyme	GTP-linked protein binding	Inhibit smooth muscle contraction
1452328_s_at	1.53	0.041	0.0018	*Pja2*	Protein binding	Ubiquitin conjugation	Ubiquitin regulation
1418429_at	1.53	0.041	0.0013	*Kif5b*	Kinesin	Increase microtubule mobility	Cell mobility
1429434_at	1.52	0.041	0.0015	*Pik3ca*	Kinase	Erk, Mapk, Akt, Rho activation	Proliferation signaling
1459973_x_at	1.51	< .001	0.0001	*Dpp4*	Peptidase	Cytokine proteolysis	Inhibit T-cell activation
1453908_at	1.51	0.041	0.0011	*Ptprb*	Phosphatase	Sodium channel regulation	Proliferation signaling
1452385_at	1.51	< .001	< .0001	*Usp53*	Enzyme	Ubiquitin-specific peptidase	Ubiquitin regulation
1420019_at	1.50	0.041	0.0015	*Tm4sf3*^b^	Tetraspanin	Integrin binding	Activation/growth signaling
1448872_at	−3.24	0.041	0.003	*Reg3g*	Growth factor	Unknown	Epithelial cell regeneration
1419684_at	−3.19	0.041	0.0042	*Ccl8*	Cytokine	Activate WBC cytokines	Immune cell activation
1418930_at	−2.99	0.041	0.0037	*Cxcl10*	Cytokine	Mast cell/monocyte chemotaxis	Inflammatory response
1438148_at	−2.81	< .001	0.0009	*Gm1960*^c^	Cytokine	Macrophage chemotaxis	Inflammatory response
1448377_at	−2.63	< .001	0.0007	*Slpi*	Peptidase inhibitor	Bind peptidase/inhibit NFκB	Inactivate leukocyte peptidase
1449401_at	−2.42	< .001	0.0009	*C1qc*	Complement protein	Complement activation	Blood cell activation
1450849_at	−2.26	0.041	0.0037	*Hnrpu*	Transport protein	Tranport snRNA to cytoplasm	RNA processing
1419128_at	−2.19	< .001	0.0008	*Itgax*	Integrin	Adhesion/signaling	Immune response
1421326_at	−2.17	0.041	0.0016	*Csf2rb1*	Transmembrane receptor	Erk, Mapk, Akt activation	Proliferation, differentiation, survival signaling
1440801_s_at	−2.10	0.041	0.0022	*Adrbk2*	Kinase	G-protein receptor activation	Activate receptor signaling
1416871_at	−2.08	0.041	0.0025	*Adam8*	Translation regulator	Metalloendopeptidase	Release of IgE low affinity receptor
1419699_at	−2.02	0.041	0.0012	*Scgb3a1*	Cytokine	Akt inhibition	Epithelial cell differentiation
1447071_at	−1.85	0.041	0.0025	*Tcf7l2*	Transcription regulator	Activate transcription	T-cell extravasation
1437864_at	−1.83	< .001	0.0001	*Adipor2*	Lipoprotein binder	Activate Ampk	Lipid metabolism
1417025_at	−1.80	0.041	0.001	*H2-Eb1*^d^	Transmembrane receptor	MHC II antigen binding	Antigen presentation
1456022_at	−1.78	0.041	0.0015	*Hipk2*	Kinase	Increase Tgfβ, Jnk transcription	Cell cycle progression signaling
1425477_x_at	−1.73	0.041	0.0008	*H2-Ab1*^e^	Transmembrane receptor	MHC II antigen binding	Antigen presentation
1446050_at	−1.71	< .001	0.0001	*Magi1*	Kinase	ATP-dependent Akt activation	Motility/adhesion signaling
1425294_at	−1.69	0.041	0.001	*Slamf8*	Transmembrane receptor	Unknown	Immune response
1450355_a_at	−1.63	0.041	0.0009	*Capg*	Protein binding	Caps actin filaments	Leukocyte motility and phagocytosis
1422725_at	−1.63	0.041	0.0014	*Mak*	Kinase	cATP/nucleotide binding	Cell differentiation signaling
1415871_at	−1.61	0.041	0.0004	*Tgfbi*	Induced protein binder	Integrin binding	Motility/adhesion signaling
1419315_at	−1.61	0.041	0.0006	*Slamf9*	Transmembrane receptor	Unknown	Immune response
1424758_s_at	−1.59	0.041	0.0012	*Serpina10*	Peptidase inhibitor	Inhibition of F10	Coagulation regulation
1433963_a_at	−1.56	0.041	0.0006	*BC032204*^f^	Enzyme	Unknown	Unknown
1429475_at	−1.56	0.041	0.0008	*Sts1*	Enzyme	Ubiquitin conjugation	Suppress T-cell signaling/endocytosis
1419721_at	−1.55	0.041	0.0005	*Gpr109b*	G-protein receptor	Inhibit cAMP	Lipid metabolism
1426324_at	−1.54	0.041	0.0006	*H2-D1*^g^	Transmembrane receptor	MHC I antigen binding	Antigen presentation
1425837_a_at	−1.53	0.041	0.0009	*Ccrn4l*	Transcription regulator	Activate RNA polymerase II	Circadian rhythm
1416985_at	−1.51	< .001	< .0001	*Sirpa*	Phosphatase	Erk, Mapk, Akt inhibition	Immune signaling
1428942_at	−1.50	0.041	0.0002	*Mt2a*	Metallic ion binder	NFκB activation	Apoptosis/survival signaling

Gene annotations are from Affymetrix GeneChip Mouse Genome 430 2.0 Array (http://www.affymetrix.com/products/arrays/specific/mouse430_2.affx).

a Mice received *in utero* ETS or filtered AIR, were OVA-tolerized through repeated aerosol exposures and then OVA-sensitized (ip OVA injections) and challenged (OVA-aerosol) as described in “Materials and Methods.”

b Tspan8.

c Cxcl2.

d Human homologue HLA-DRB1.

e Human homologue HLA-DQB2.

f Urp2.

g Human homologue HLA-C.
